# Golgi-Resident GTPase Rab30 Promotes the Biogenesis of Pathogen-Containing Autophagosomes

**DOI:** 10.1371/journal.pone.0147061

**Published:** 2016-01-15

**Authors:** Seiichiro Oda, Takashi Nozawa, Atsuko Nozawa-Minowa, Misako Tanaka, Chihiro Aikawa, Hiroyuki Harada, Ichiro Nakagawa

**Affiliations:** 1 Department of Bacterial Pathogenesis, Graduate School of Medical and Dental Science, Tokyo Medical and Dental University, Tokyo 113–8549, Japan; 2 Department of Oral and Maxillofacial Surgery, Graduate School of Medical and Dental Science, Tokyo Medical and Dental University, Tokyo 113–8549, Japan; 3 Department of Microbiology, Graduate School of Medicine, Kyoto University, Yoshida-Konoe-cho, Sakyo-ku, Kyoto 606–8501, Japan; Institute of Molecular and Cell Biology, Biopolis, UNITED STATES

## Abstract

Autophagy acts as a host-defense system against pathogenic microorganisms such as Group A *Streptococcus* (GAS). Autophagy is a membrane-mediated degradation system that is regulated by intracellular membrane trafficking regulators, including small GTPase Rab proteins. Here, we identified Rab30 as a novel regulator of GAS-containing autophagosome-like vacuoles (GcAVs). We found that Rab30, a Golgi-resident Rab, was recruited to GcAVs in response to autophagy induction by GAS infection in epithelial cells. Rab30 recruitment was dependent upon its GTPase activity. In addition, the knockdown of Rab30 expression significantly reduced GcAV formation efficiency and impaired intracellular GAS degradation. Rab30 normally functions to maintain the structural integrity of the Golgi complex, but GcAV formation occurred even when the Golgi apparatus was disrupted. Although Rab30 also colocalized with a starvation-induced autophagosome, Rab30 was not required for autophagosome formation during starvation. These results suggest that Rab30 mediates autophagy against GAS independently of its normal cellular role in the structural maintenance of the Golgi apparatus, and autophagosome biogenesis during bacterial infection involves specific Rab GTPases.

## Introduction

Autophagy is an intracellular bulk degradation system that is induced by nutrient deprivation. In the initial step of autophagy, a part of the cytoplasm is non-selectively encapsulated by an isolation membrane. Isolation membranes are fused at their edges to form double membrane vesicles, known as autophagosomes. Subsequently, autophagosomes fuse with lysosomes, eventually leading to degradation of the sequestered contents by lysosomal enzymes [1–3]. Furthermore, autophagy is also executed in a selective manner against pathogenic microbes, such as Group A *Streptococcus* (GAS), *Listeria monocytogenes*, *Salmonella enterica* serovar Typhimurium, and viruses, in a process termed xenophagy [4–6].

GAS is a common pathogen that causes a variety of acute infections including pharyngitis, skin infections, acute rheumatic fever, and life-threatening necrotizing fasciitis [7]. GAS enters non-phagocytic human cells via endocytosis but escapes from endosomal membranes through the activity of streptolysin O (SLO), a pore-forming toxin secreted by GAS [8]. GAS in cytoplasm are targeted by the ubiquitin-p62/NDP52 pathway and LC3-positive autophagic membrane structures [8,9], termed GAS-containing autophagosome-like vacuoles (GcAVs). Each GcAV coalesces into a large GcAV via Rab7 [10]. GcAVs acquire lysosomal enzymes through fusion with lysosomes, and GAS is then degraded in this autolysosome [11–13].

The complex membrane dynamics involved in generating autophagosomes under starvation conditions have been extensively investigated. Previous studies have demonstrated that autophagic vacuole formation involves a number of membrane traffic regulators including Rab GTPases, which act as molecular switches to regulate vesicular traffic [14,15]. To date, Rab1A, Rab1B, Rab4, Rab5, Rab7, Rab8B, Rab11, Rab24, and Rab33B have been implicated in starvation-induced autophagosome formation processes [14,16,17]. We have investigated the role of Rab proteins in GcAV formation and found that the Rab proteins that regulate GcAV processes are largely distinct from those that act during starvation-induced autophagy [18,19]. However, the Rab proteins identified as regulators of GcAV include only Rab7, Rab9A, Rab17, and Rab23. Thus, more information is necessary to understand the dynamic mechanical behaviors of autophagosomes in response to GAS infection.

While screening for Rab proteins that localize to GcAVs by confocal microscopy colocalization analysis, we found that emerald green fluorescent protein (EmGFP)-labeled Rab30 was visible on most GcAVs. Rab30 is a ubiquitously expressed Rab protein and has been shown to be primarily associated with the Golgi [20]. Rab30 also associates with a number of golgin proteins in *Drosophila melanogaster*, the fly orthologues of the coiled-coil proteins p115 and Bicaudal-D [21]. A recent study has shown that Rab30 function is required for the structural integrity of the Golgi apparatus in HeLa cells [22]. Rab30 is also known to be a target of the jun-N-terminal kinase (JNK), a regulator of gene expression, and suggested to be involved in head involution and thorax fusion during *D*. *melanogaster* development [23]. However, neither the involvement of Rab30 in autophagy nor the functions of Rab30 during pathogenic infection have not been addressed. In this study, we examined the subcellular localization of Rab30 during GAS infection in detail, its role in autophagy against GAS, and identified Rab30 as a new regulator of GcAV formation.

## Materials and Methods

### Cell culture and transfections

HeLa cells line was purchased from the American Type Culture Collection and maintained in Dulbecco’s Modified Eagle’s Medium (DMEM) (Nalacai Tesque) supplemented with 10% fetal bovine serum (FBS) (JRH Biosciences) and 50 μg/mL gentamicin (Nacalai Tesque) in a 5% CO_2_ incubator at 37°C. To induce starvation, cells were incubated in Hanks’ balanced salt solution (starvation medium) (Nacalai Tesque). Plasmid transfections were performed using polyethylenimine (Polyscience) or Lipofectamine 3000 (Invitrogen), according to the manufacturers’ protocols.

### GAS strain

GAS strain JRS4 (M6^+^ F1^+^) was grown in Todd–Hewitt broth (BD Diagnostic Systems, Sparks, MD) supplemented with 0.2% yeast extract (THY), as described previously [12].

### Plasmids

Gateway cloning technology (Invitrogen) was used to create the vectors indicated below. Human Rab30 protein cDNA (GenBank Accession No. NM_001286059.1) was amplified from human cDNA libraries using the following primer pair: Rab30_F, 5′-CACCATGAGTATGGAAGATTATGATTTCCTGTTCAA-3′, and Rab30_R, 5′-GCCTTTAGTTGAAATTACAACAAGTCAAATAGCTGA-3′. The PCR product was cloned into the pENTR/D-TOPO vector using the pENTR Directional TOPO Cloning Kit (Invitrogen) and subcloned into the pcDNA6.2/N-EmGFP-DEST or pcDNA6.2/N-mCherry-DEST vector. Constitutively negative Rab30 (Rab30 T23N) and active (Rab30 Q68L) mutants were constructed by introducing a point mutation using the following primer pairs: Rab30_T23N_F, 5′-AACGCTGGTGTGGGGAAGAACTGCCTC-3′, and Rab30_T23N_R, 5′- GAATCTTCGGACGAGGCAGTTCTTCCC-3′; Rab30_Q68L_F, 5′- ATCTGGGACACAGCAGGTTTAGAGAGA-3′ and Rab30_Q68L_R, 5′- AATGGACCGAAATCTCTCTAAACCTGC-3′. Small interfering RNAs (siRNAs) against Rab30 (#1: 5′-AAGAGAGAUUUCGGTCCAUUA-3′, #2: 5′-GCAUUAGCAGAACAUAUAA-3′; HP custom siRNA hRab30, Qiagen), Rab1 (Hs_RAB1A_4, Qiagen), Arf1 (Hs_ARF1_1, Qiagen), and a control siRNA target (FlexiTube siRNA SI03650318, Qiagen) were used for silencing experiments.

### Reagents

Brefeldin A and Golgicide A were purchased from Nacalai Tesque and Calbiochem, respectively.

### GAS infection

Infections with GAS were performed as described previously [12]. Bacterial titers were determined by measuring colony-forming units (CFUs). GAS grown through mid-log phase was added to cell cultures at a multiplicity of infection (MOI) of 100, without antibiotics. After 1 h, infected cells were washed with phosphate-buffered saline (PBS), and then 10% DMEM/FBS with antibiotics (100 μg/mL gentamicin) was added to kill extracellular bacteria. The cells were further cultured for the indicated times.

### Antibodies

The following antibodies were used: mouse monoclonal anti-FLAG M2 (Sigma-Aldrich), mouse monoclonal anti-ubiquitinated protein (FK2; Nippon Bio-Test Laboratories), mouse monoclonal anti-Rab30 (Abcam, ab156774), rabbit monoclonal anti-Atg5 (Cell Signaling Technology, #4967), rabbit monoclonal anti-β-actin (Cell Signaling Technology, #4970), mouse monoclonal anti-LAMP1 (Santa Cruz, sc-20011), and rabbit monoclonal anti-GM130 (BD Transduction Laboratories, 610822). The secondary antibodies used for immunoblotting were horseradish peroxidase-conjugated anti-mouse or anti-rabbit IgG (Jackson Immunoresearch Laboratories). The fluorescent secondary antibodies used for immunofluorescence were Alexa Fluor 488-conjugated goat anti-mouse or anti-rabbit IgG, Alexa Fluor 594-conjugated goat anti-mouse or anti-rabbit IgG, or Alexa Fluor 660-conjugated goat anti-mouse or anti-rabbit IgG (Molecular Probes/Invitrogen).

### Fluorescence microscopy

For immunostaining experiments, cells were washed with PBS, fixed with 4% paraformaldehyde in PBS for 15 min, and permeabilized with 0.1% Triton in PBS for 5 min. The cells were blocked with skim milk blocking solution (5% skim milk, 2.5% goat serum, and 2.5% donkey serum in PBS containing 0.1% gelatin) or BSA blocking solution (2% BSA and 0.02% sodium azide in PBS) for 1 h and incubated with primary antibodies in blocking solution at room temperature for 1 h. After washing with PBS, the cells were then probed with secondary antibodies for 1 h. To label bacterial and cellular DNA, cells were stained with 4,6-diamidino-2-phenylindole (DAPI) (Nacalai Tesque) in blocking solution. All fluorescent confocal microscopy images were acquired with an FV1000 laser-scanning microscope (Olympus).

### Bacterial viability assays

HeLa cells transfected with control siRNA or Rab30 siRNA were cultured in 24-well plates and infected with GAS as described above. After an appropriate incubation time, infected cells were washed with PBS. Infected cells were disrupted in distilled water, serial dilutions of the lysates were plated on THY agar plates, and colony counting was performed. Recovered bacteria were calculated by measuring CFUs at 6 h post-infection divided by CFUs at 2 h post-infection.

### Generation of knockout lines using CRISPR/Cas9 gene editing

In order to construct a Atg5 knockout cell line, the CRISPR/Cas9 system [24] was used. CRISPR guide RNAs (gRNAs) were chosen that targeted an exon common to all splicing variants of the gene of interest (target sequence; ATCAAGTTCAGCTCTTCCT). To select transfected cells with antibiotic, we constructed gRNA vector carrying hygromycin resistance gene (gRNA-hyg vector); we cloned gRNA cassette (U6 promoter-gRNA scaffold-termination signal sequence) of gRNA vector (Addgene 418284) into pcDNA3.1hygro(-) in which original CMV promoter was removed beforehand. Oligonucleotides containing CRISPR target sequences were annealed and ligated into AlfII-linearized gRNA-hyg vector. For CRISPR/Cas9 gene editing, HeLa cells were transfected with gRNA-hyg constructs and hCAS9 (Addgene 41815). Two days after transfection, untransfected cells were removed by selection with 300 μg/mL Hygromycin B (Nacalai Tesque) and 750 μg/mL Geneticin (G418) (Nacalai Tesque). Single colonies were expanded into 24-well plates before screening for depletion of the targeted gene product by immunoblotting. As a secondary screen of some knockout lines, genomic DNA was isolated from cells and the genomic regions of interest were amplified using PCR. Sequencing of targeted genomic regions of knockout lines was also conducted to confirm the presence of frameshifting indels in the genes of interest.

### Statistical analysis

Quantification of colocalization and GcAV formation was performed by direct visualization with confocal microscopy. Unless otherwise indicated, at least 50 GcAVs or 200 GAS-infected cells were counted per condition for each experiment. For all graphs, at least three independent experiments were performed. Unless otherwise indicated, the mean ± standard deviation (SD) is shown and *p*-values were calculated using a two-tailed Student’s *t*-test. A *p*-value of less than 0.05 was determined to be statistically significant.

## Results

### Localization of Rab30 during GAS infection

**Fig 1:**
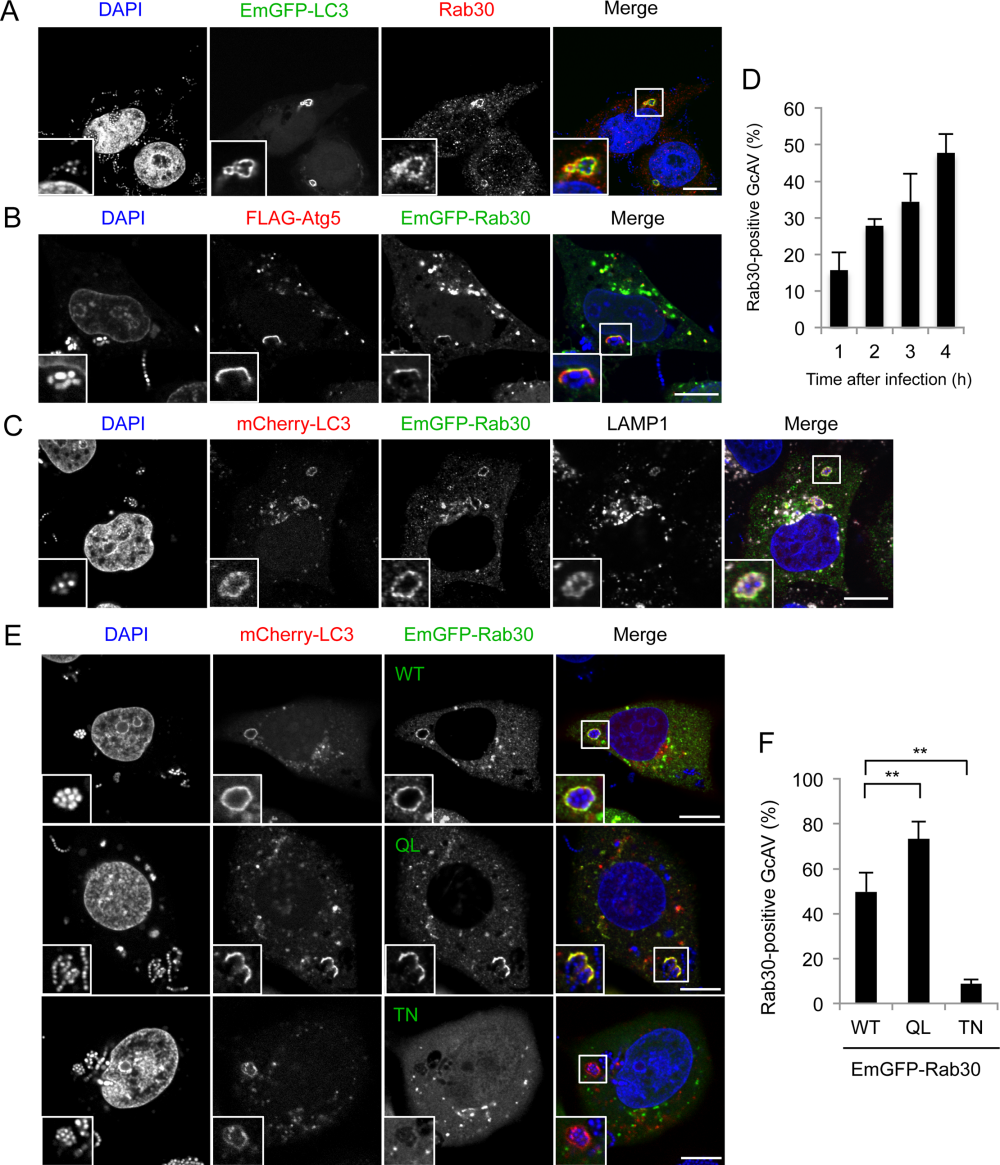
Golgi-resident GTPase Rab30 is localized to GAS-targeting autophagic structures. (A) HeLa cell transiently expressing EmGFP-LC3 were infected with GAS (MOI = 100) for 4 h. Cells were then fixed, permeabilized, and immunostained with an anti-Rab30 antibody. Cellular and bacterial DNA was stained with DAPI. (B) HeLa cells transiently expressing FLAG-Atg5 and EmGFP-Rab30 were infected with GAS (MOI = 100) for 2 h. Cells were then immunostained with an anti-FLAG antibody. (C) HeLa cells transiently expressing mCherry-LC3 and EmGFP-Rab30 were infected with GAS for 4 h. Cells were then immunostained with an anti-LAMP1 antibody. Bars, 10 μm. (D) The number of cells containing GcAVs were counted and presented as the percentage of the total number of GAS-infected cells. Cells were transfected and infected with GAS for 4 h, as described in (C). The data shown represent result from >200 infected cells in terms of the mean value ± SD from 3 independent experiments. (E) HeLa cells transiently expressing mCherry-LC3 and EmGFP-Rab30 WT, EmGFP-Rab30 Q68L (QL), or EmGFP-Rab30 T23N (TN) were infected with GAS for 4 h. Bars, 10 μm. (F) Colocalization frequencies of GcAV and Rab30 were counted and presented as the percentage of the total number of GcAVs. The data shown represent the result from >80 GcAVs in terms of the mean value ± SD from 3 independent experiments. ** *P* < 0.01.

In previous screening experiments [18,19], we found that EmGFP-Rab30 colocalizes with GcAV. To confirm the GcAV localization of Rab30, we examined the subcellular localization of endogenous Rab30 during GAS infection. Rab30 was found localized around GAS and colocalized with GcAV (Fig 1A). To examine Rab30 localization during GAS infection in more detail, we transiently expressed EmGFP-Rab30 and mCherry-LC3 (a red fluorescent protein fused to an autophagic membrane marker) or FLAG-Atg5, and infected cells with GAS. Previously, we demonstrated that Atg5 localizes specifically to the isolation membrane and can be used as an isolation membrane marker protein during GAS infection [18]. We found that EmGFP-Rab30 colocalized with GAS-associated Atg5-positive membranes (Fig 1B), suggesting that Rab30 is localized to GAS-targeting isolation membranes. In addition, EmGFP-Rab30 clearly overlapped with LAMP1-positive GcAV at 4 h after infection (Fig 1C). These results suggested that Rab30 localizes to GcAVs from isolation membranes to lysosome-fused GcAV autolysosome. Next, we examined the time course of colocalization of Rab30 with GcAV. As shown in Fig 1D, colocalization increased gradually over time during GAS infection, suggesting that Rab30 is recruited to GcAV and accumulated on the GcAV membranes.

Rab GTPases function as molecular switches by cycling between GTP-bound and GDP-bound states. To examine whether the recruitment of Rab30 to GcAVs is determined by the GTP/GDP-bound status, we constructed EmGFP-Rab30 Q68L (constitutively active, GTP-bound form) and EmGFPRab30 T23N (constitutive negative, GDP-bound form). EmGFP-Rab30 Q68L showed significantly higher colocalization with GcAVs than did EmGFP-Rab30 WT (** *P* < 0.01), whereas EmGFP-Rab30 T23N was rarely detected on GcAVs (Fig 1E and 1F). These results suggest that the recruitment of Rab30 to GcAVs depends upon GTP binding.

### Recruitment of Rab30 to GcAVs

**Fig 2:**
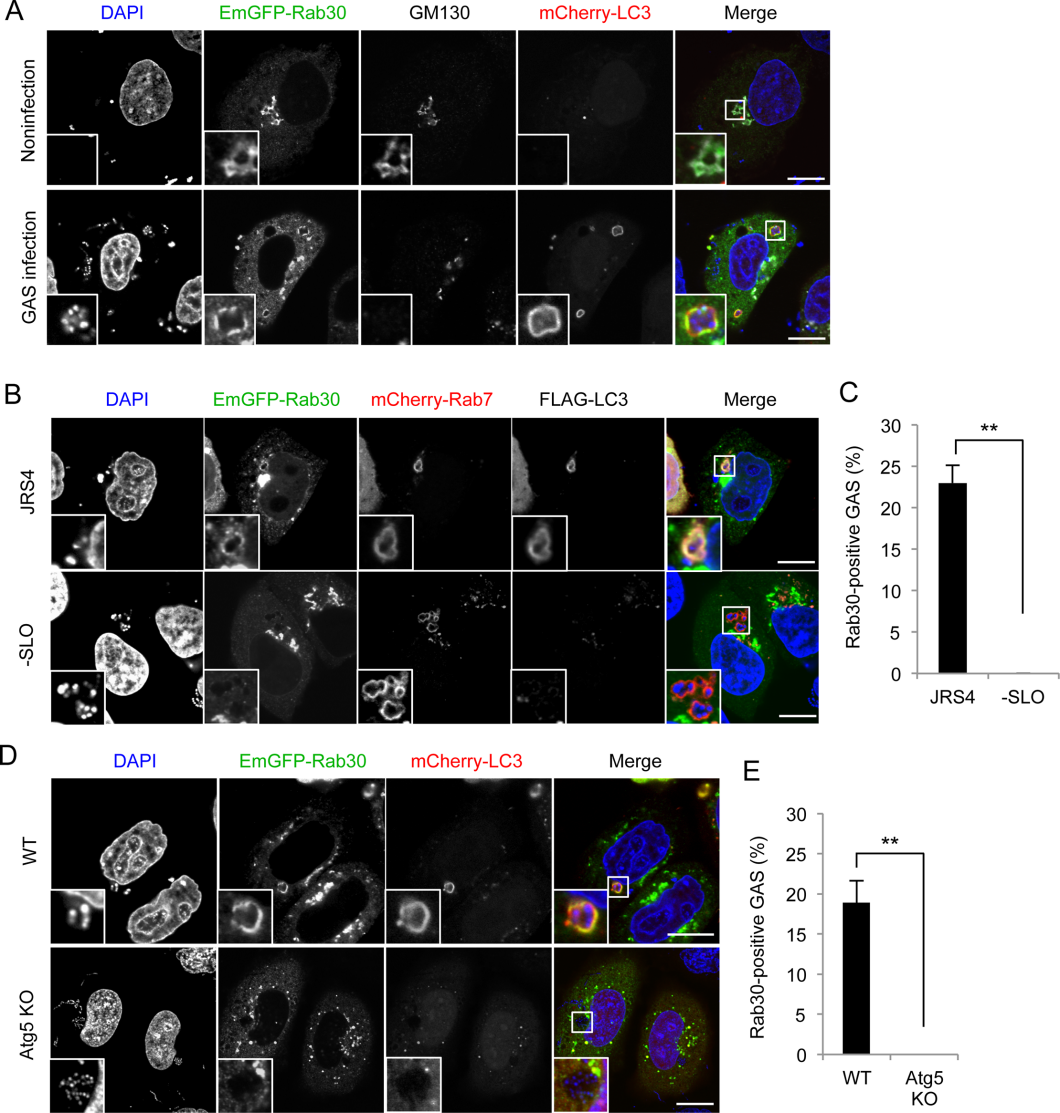
Rab30 is redistributed from the Golgi apparatus to GcAVs upon autophagy induction by GAS. (A) HeLa cells transiently expressing mCherry-LC3 and EmGFP-Rab30 were infected with GAS (MOI = 100) for 4 h. Cells were immunostained with an anti-GM130 antibody. (B) HeLa cells transiently expressing EmGFP-Rab30, mCherry-Rab7, and FLAG-LC3 were infected with JRS4 WT or JRS4ΔSLO for 4 h. (C) The percentages of cells with Rab30-associated GAS were quantified. Data represent the result of >100 cells in terms of the mean value ± SD from 3 independent experiments. (D) HeLa WT or Atg5 knockout (KO) cells transiently expressing EmGFP-Rab30 and mCherry-LC3 were infected with GAS for 4 h. Bars, 10 μm. (E) The percentages of cells with Rab30-associated GAS were quantified. ** *P* < 0.01.

Rab30 is primarily associated with the Golgi complex, which is considered to be a potential membrane source for autophagy [22,25]. To examine the possibility that Rab30 localizes to GcAVs as a secondary consequence of Golgi-derived membrane incorporation into GcAVs, we studied the colocalization of Rab30 and LC3 with the Golgi apparatus during GAS infection. In uninfected cells, EmGFP-Rab30 clearly colocalized with GM130, a *cis*-Golgi marker protein, while the mCherry-LC3 signal was present diffusely in the cytoplasm with a few punctate dots that did not colocalize with EmGFP-Rab30 (Fig 2A). However, in GAS-infected cells, mCherry-LC3 was found around GAS and clearly colocalized with EmGFP-Rab30, and the LC3-positive structure did not contain GM130 (Fig 2A). These results suggest that Rab30 does not localize to GcAVs via Golgi-derived membrane fusion, but is redistributed to GcAVs instead.

Internalized GAS are exposed to the cytosol through SLO-mediated disruption of endosomes, and these bacteria are targeted by autophagy via an ubiquitin-adaptor pathway [12]. SLO-deficient GAS were rarely surrounded by galectin 8, which is a cytosolic lectin and recruited to damaged-endosome [26], in HeLa cells (S1A and S1B Fig). Moreover, recruitment of ubiquitin and GcAV formation were rarely observed when SLO-deficient GAS were infected (S1C, S1D and S1E Fig). Then, to examine whether Rab30 is recruited to intracellular GAS in response to autophagy induction, we infected SLO-deficient GAS and observed the localization of Rab30, LC3, and Rab7 that can localize to endosomes and GcAV [10]. As shown in Fig 2B and 2C, EmGFP-Rab30 was not recruited to SLO-deficient GAS which are Rab7-positive intraendosomal bacteria, suggesting that Rab30 is recruited to GAS in response to autophagy induction by SLO.

We also examined whether the recruitment of Rab30 to GAS requires GcAV formation using autophagy-deficient cell line. In order to generate autophagy-deficient HeLa cells, genome editing was used to knockout (KO) Atg5, which is essential for autophagy. Immunoblotting of Atg5 confirmed its knockout (S2A Fig), and confocal microscopy analysis revealed that Atg5 is essential for GcAV formation in HeLa cells (S2B and S2C Fig). We then investigated the localization of Rab30 in Atg5 KO HeLa cells infected with GAS. As expected, recruitment of EmGFP-Rab30 into GAS invaded HeLa cells was impaired by knockout of Atg5 (Fig 2D and 2E), indicating that the Rab30 localization to GAS is dependent on GcAV formation.

### Involvement of Rab30 in GcAV formation during GAS infection

**Fig 3:**
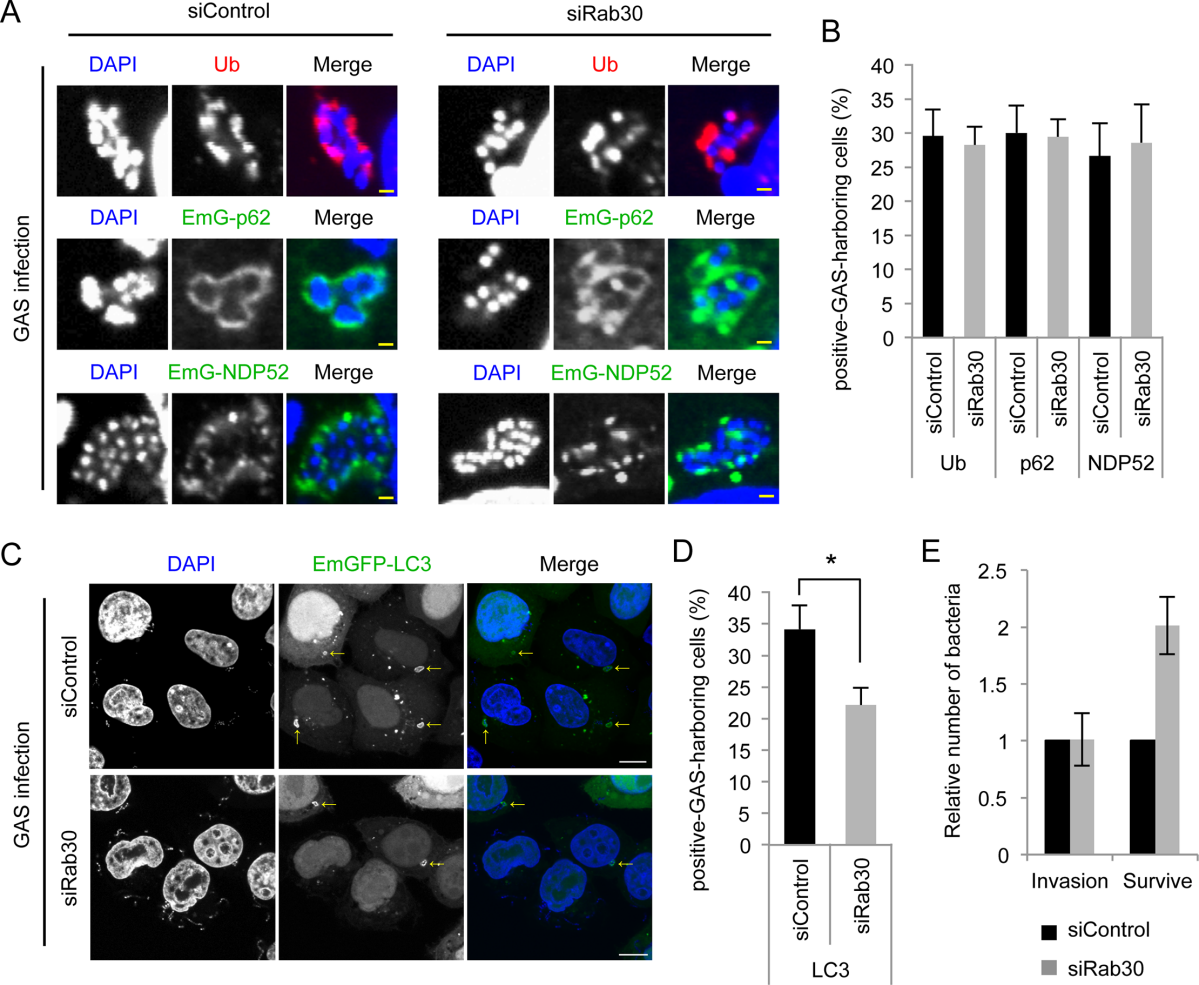
Rab30 is involved in autophagy during GAS infection. (A) HeLa cells were transfected with a control siRNA or Rab30 siRNA (siRab30 #1), as well as mCherry-LC3, and EmGFP-p62, or EmGFP-NDP52 expression vector and then infected with GAS for 4 h. Cells were then fixed, permeabilized, and immunostained with an anti-ubiquitinated protein (Ub) antibody. Yellow bars, 2 μm. (B) The number of cells containing Ub/NDP52/p62-positive GAS were counted and presented as the percentage of the total number of GAS-infected cells. Data represent the results of >100 cells in terms of the mean value ± SD from 3 independent experiments. (C) HeLa cells transfected with control siRNA or Rab30 siRNA (siRab30 #1), as well as an EmGFP-LC3 expression vector were infected with GAS for 4 h. The cells were then fixed, permeabilized, and stained with DAPI. Yellow arrows indicate GcAV. Bars, 10 μm. (D) The number of cells containing GcAVs was counted and presented as the percentage of the total number of GAS. Data represent the results of >100 cells in terms of the mean value ± SD from 3 independent experiments. * *P* < 0.05. (E) HeLa cells were transfected with control or Rab30 siRNA (siRab30 #1). At 48 h post-transfection, HeLa cells were infected with GAS for 1, 2, or 6 h. Recovered bacteria were measured in GAS viability assays. The data shown represent the mean value ± SD from 3 independent experiments.

To clarify the function of Rab30 in GAS-induced autophagy, we investigated the involvement of Rab30 in the recruitment of autophagy-related molecules to GAS. We first tried to generate Rab30 knockout HeLa cells using CRISPR/Cas9 genome editing system, but we could not obtain the knockout cell. Then, we used siRNAs to knockdown Rab30 expression [22] and confirmed the knockdown effect of these siRNAs in HeLa cells by immunoblotting and immunostaining (S3A and S3B Fig). As mentioned above, GAS invasion into the cytoplasm induces autophagy, and cytoplasmic GAS becomes ubiquitin (Ub)-positive, which facilitates targeting of autophagosome to GAS via cargo receptors [9,27]. We then examined the percentage of Ub, NDP52, p62, or LC3-positive infected cells. As shown in Fig 3A and 3B, GAS was coated with Ub, NDP52, and p62, and the percentages of positive cells were not changed by silencing Rab30 expression, suggesting that invasion events, i.e., endocytosis of GAS, endosomal disruption by GAS, and recruitments of recognition molecules to GAS were not affected by Rab30 knockdown. However, silencing Rab30 expression significantly decreased the percentage of GcAV-harboring cells compared with control cells (*P* < 0.05) (Fig 3C and 3D). To confirm this effect of Rab30 knockdown, we used additional siRNA that target UTR of Rab30 (siRab30 #2). The GcAV formation efficiency was significantly decreased by Rab30 knockdown using siRab30#2 (S3C and S3D Fig). Taken together, this suggests that Rab30 is not required for the recognition step, but is involved in GcAV biogenesis.

We next investigated the effects of Rab30 WT, QL and TN overexpression on GcAV formation. Overexpression of Rab30 QL increased the GcAV formation efficiency while Rab30 TN overexpression significantly decreased the percentage of GcAV-positive cells compared with control or Rab30 WT overexpressing cells (S4A and S4B Fig), thereby suggesting that GTP-bound form of Rab30 is involved in GcAV formation.

Next, to ascertain whether the reduction in GcAVs in Rab30-knockdown cells affects the intracellular growth of GAS, we determined the number of surviving bacteria in control and Rab30-knockdown cells. Invading bacteria that survived were counted in colony-formation assays (bacteria viability assay). Although Rab30 knockdown did not affect GAS invasion, the survival rate of GAS at 6 h post-infection was significantly higher in Rab30-knockdown cells than in control cells (Fig 3E). Taken together, these results demonstrated that Rab30 is involved in the autophagic killing of GAS.

### The role of Rab30 in GAS autophagy does not depend on its function in maintaining the structural integrity of Golgi apparatus

**Fig 4:**
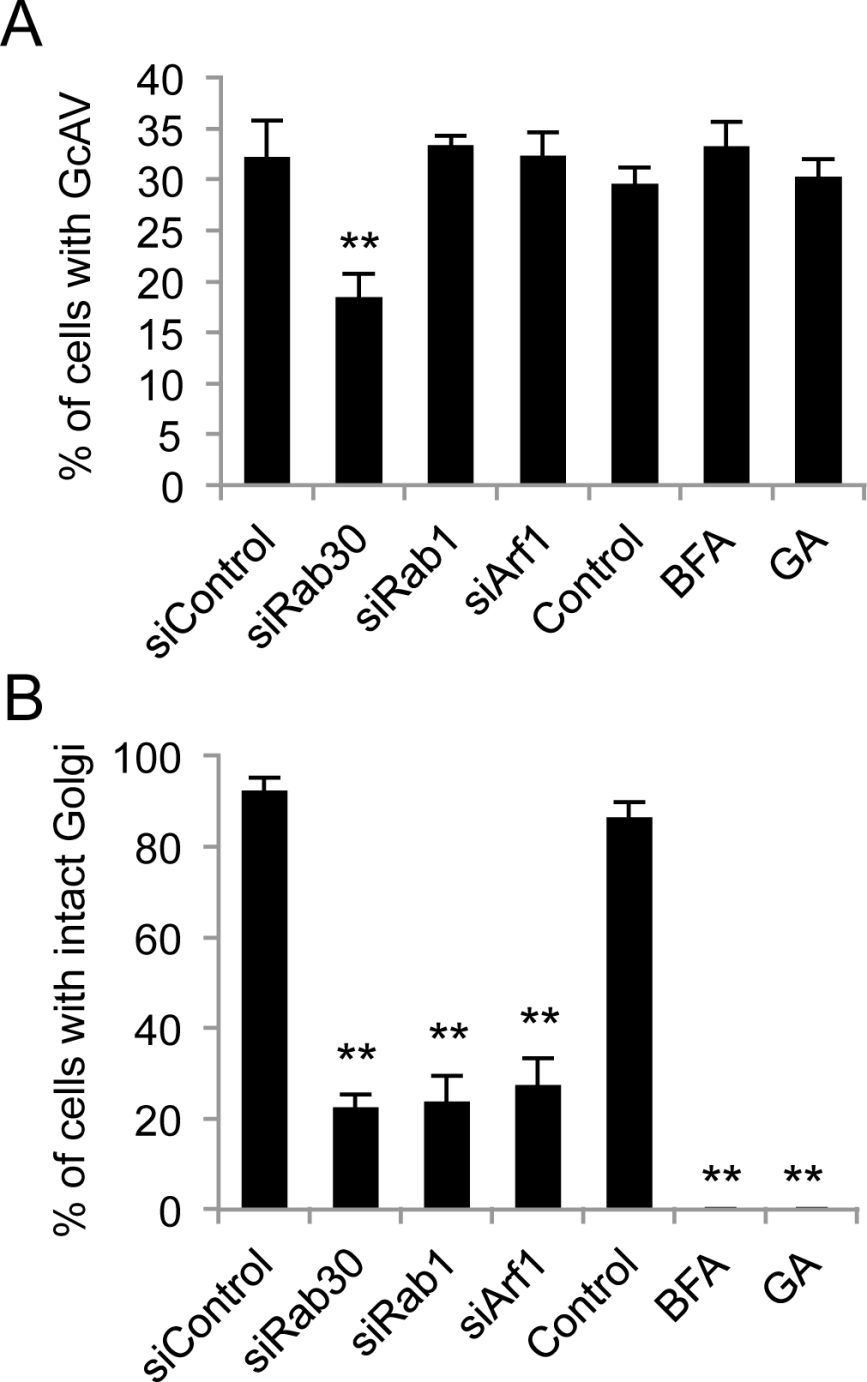
Involvement of Rab30 in GAS-induced autophagy is independent of its role in maintaining the structural integrity of the Golgi apparatus. (A) HeLa cells were transfected with EmGFP-LC3 and siRNAs against the indicated target mRNAs, or were treated with BFA or GA, as indicated. Subsequently, the cells were infected with GAS (MOI = 100). The cells were then fixed, permeabilized, and stained with DAPI. The percentage of cells with GcAVs was quantified. (B) HeLa cells were transfected with siRNAs against indicated target mRNAs, or treated with BFA or GA, after which they were infected with GAS. Cells were fixed, permeabilized, and immunostained with an anti-GM130 antibody. The percentage of cells with intact Golgi structure was quantified. The data shown represent the results of >200 cells in terms of the mean value ± SD from 3 independent experiments. ** *P* < 0.01.

Rab30 is reported to play a crucial role in maintaining the structural integrity of the Golgi complex, and Rab30 knockdown causes disruption of the Golgi apparatus [22]. Although we did not observe the recruitment of Golgi-membrane markers into GcAVs, we cannot exclude the possibility that the Golgi complex is involved in GcAV formation and that the impairment of GcAV formation in Rab30-knockdown cells results from destruction of the Golgi structure. To examine this possibility, we silenced Rab1 and Arf1 expression and examined the GcAV formation and the morphology of the Golgi complex. Although depletion of Rab30, Rab1, or Arf1 caused Golgi fragmentation, only Rab30 knockdown reduced the GcAV-formation efficiency (Fig 4A and 4B). Moreover, treatment of HeLa cells with Brefeldin A (BFA) or Golgicide A prior to GAS infection did not affect GcAV formation, even though the Golgi was disrupted (Fig 4A and 4B). These results suggest that disruption of the Golgi apparatus has little impact on GcAV formation and that the involvement of Rab30 in GcAV formation is independent of its normal cellular function in maintaining the structural integrity of the Golgi apparatus.

### Rab30 is dispensable for starvation-induced autophagosome formation

**Fig 5:**
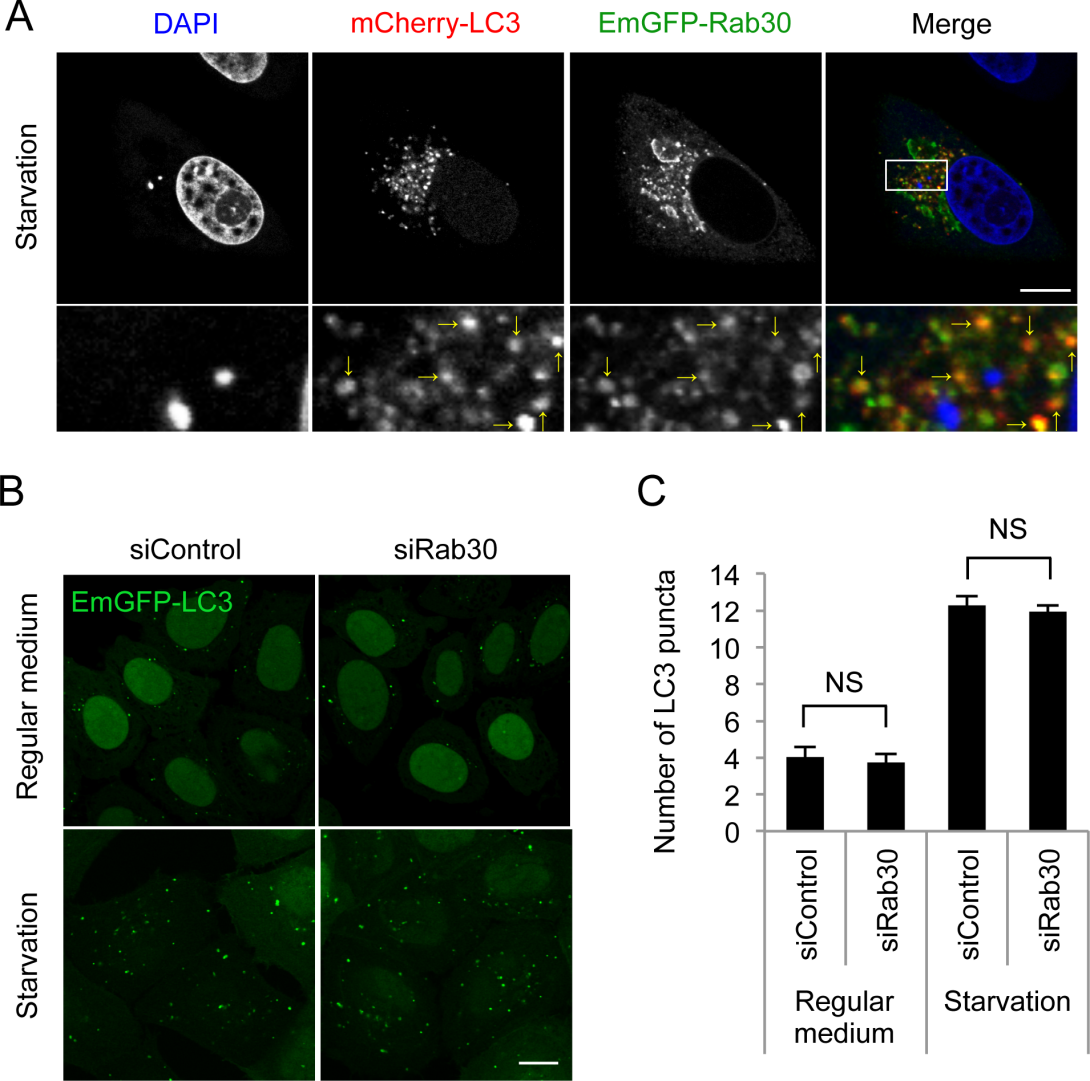
Involvement of Rab30 in starvation-induced autophagosome formation. (A) Confocal microscopic images of mCherry–LC3 puncta with EmGFP–Rab30 in starvation conditions. HeLa cells that expressed mCherry–LC3 and EmGFP–Rab30 were cultured in starvation medium for 2 h and fixed. Yellow arrows indicate Rab30-colocalized LC3 puncta. (B) Confocal microscopic images of EmGFP–LC3 puncta in Rab30 knockdown cells. HeLa cells stably expressing EmGFP–LC3 were transfected with a control siRNA or Rab30 siRNA and cultured in regular medium or starvation medium for 2 h and fixed. Bars, 10 μm. (C) Effect of knockdown of the Rab30 on starvation-induced autophagosome formation. The number of LC3 dots was quantified in confocal microscopic images. The data shown represent the results for >10 images and each percentage represents the mean value ± SD based on three independent experiments. NS, not significant.

To further examine whether Rab30 is involved in starvation-induced autophagosome formation, we investigated the colocalization of EmGFP-Rab30 with mCherry-LC3 in starvation conditions. The LC3-positive puncta efficiently colocalized with EmGFP-Rab30 (Fig 5A), suggesting the involvement of Rab30 in starvation-induced autophagy. However, the numbers of LC3 puncta under nutrient and starvation condition were not affected by Rab30 knockdown (Fig 5B and 5C). Therefore, it is suggested that Rab30 is not required for autophagosome formation during starvation and GAS targeting autophagy involves different Rab proteins-mediated mechanisms.

## Discussion

In the present study, we identified Rab30 as a regulator for autophagy against GAS infection. The results of our study suggest that Rab30 is recruited to GcAVs and facilitates GcAV formation. To date, the roles of only 4 Rab proteins (Rab7, Rab9A, Rab17, and Rab23) in GAS-induced autophagy have been reported [10,18,19,28]. At the early stage of GcAV formation, Rab7 and Rab23 function to form GcAVs, and Rab9A mediates autophagosome-lysosome fusion at the late stage of autophagy. Knockdown analyzes of Rab7 and Rab23 functions have suggested that Rab7 is required for homotypic fusion between isolation membranes to form GcAVs and that Rab23 is involved in targeting isolation membranes to intracellular GAS. Rab17 has been reported to function to supply membrane from recycling endosomes to GcAVs. In this study, it has also been suggested that Rab30 regulates GcAV formation, but its precise role in GcAV formation remains unclear. Both Rab7 and Rab23 are endosome-resident Rab GTPases that regulate endosome-lysosome fusion in non-infected cells [29]. In contrast, Rab30 localizes to the Golgi complex and functions in the structural maintenance of the organelle, and its involvement in GcAV formation was independent of Golgi structural maintenance. Interestingly, a recent study involving a comprehensive screen of Rab effectors reported that Rab30 associates with not only Golgi-component proteins but also the exocyst complex [30]. The exocyst complex has been shown to regulate autophagy induction through the associations with ULK1 and Beclin 1 [31]. Therefore, Rab30 might regulate post-Golgi trafficking, and the Rab30-mediated pathway might be involved in GcAV formation.

In canonical autophagy, several Rab proteins are redistributed to autophagic membranes from various organelles (endosomes, recycling endosomes, the Golgi complex, the endoplasmic reticulum, and intermediate vesicles) and participate in autophagosome formation [14,15]. However, Rab proteins on GcAVs (Rab7, Rab9A, Rab17, and Rab23) are all related to endosomal compartments and are largely distinct from Rab proteins acting in starvation-induced autophagy. Because GAS is internalized through an endosomal pathway, this defensive pathway against GAS invasion may reflect a product of evolutionary pressure. We found that Rab30 was redistributed to autophagosomes from the Golgi apparatus during GAS infection. This is the first report that Golgi-related Rab30 associates with the LC3-positive compartment, and the present data suggest an importance of membrane dynamics other than endocytosis during autophagy against bacteria.

Our data also showed that the recruitment of Rab30 to GAS-associated LC3-positive membranes was dependent upon the GTP-bound form and that Rab30-positive GcAVs are defective in Golgi-marker proteins. As the nucleotide cycle is coupled to membrane attachment and release, the localization of the guanine nucleotide-exchange factor (GEF) and the GTPase activating protein (GAP) are suggested to determine the correct targeting of Rab proteins [32]. Thus far, neither a GEF nor a GAP for Rab30 has been identified. Thus, identifying the GEF and GAP for Rab30 would be informative in understanding how host cells respond to bacterial infection and regulate autophagic vacuole formation.

In conclusion, we identified Rab30 as a novel regulator of autophagy against GAS. The involvement of Rab30 in GcAV formation is likely independent of its roles in the structural maintenance of Golgi apparatus. Because knowledge regarding the role of Rab30 is limited with respect to both pathogen-infected and uninfected cells, additional investigations on Rab30 functions will help understand the host-defense system and autophagic membrane trafficking.

## Supporting Information

S1 Fig Localizations of autophagy-related markers in SLO-deficient GAS-infected HeLa cells. (A) Confocal microscopic images of EmGFP–Galectin-8 (Gal-8) with mCherry–LC3 in SLO-deficient GAS-infected cells. HeLa cells that expressed mCherry–LC3 and EmGFP–Gal-8 were infected with WT or SLO-deficient GAS for 4 h. Cells were then fixed, permeabilized, and stained for cellular and bacterial DNA with DAPI. Bars, 10 μm. (B) The percentages of cells with EmGFP-Gal-8-associated GAS were quantified. Data represent the result of >100 cells in terms of the mean value ± SD from 3 independent experiments. (C) Confocal microscopic images of EmGFP–LC3 and ubiquitin in SLO-deficient GAS-infected cells. HeLa cells that expressed EmGFP–LC3 were infected with WT or SLO-deficient GAS for 4 h. Cells were then fixed, permeabilized, and stained with anti-ubiquitin (Ub) antibody. (D, E) The percentages of cells with ubiquitin- (D) or LC3- (E) associated GAS were quantified. Data represent the result of >100 cells in terms of the mean value ± SD from 3 independent experiments. ** *P* < 0.01.
(TIF)

S2 Fig Atg5 is essential for GcAV formation in HeLa cells. (A) HeLa WT or Atg5 knockout (KO) cells were analyzed by immunoblotting using anti-Atg5 antibody. (B) Confocal microscopic images of EmGFP–LC3 in Atg5 KO cells during GAS infection. HeLa WT or Atg5 KO cells that expressed EmGFP–LC3 were infected with GAS for 4 h. Cells were then fixed, permeabilized, and stained for cellular and bacterial DNA with DAPI. Bars, 10 μm. (D) The percentages of cells harboring GcAVs were quantified. Data represent the result of >200 cells in terms of the mean value ± SD from 3 independent experiments. ** *P* < 0.01.
(TIF)

S3 Fig Silencing of Rab30 expression by siRNA. (A) HeLa transfected with siControl or siRab30 were analyzed by immunoblotting using anti-Rab30 antibody. (B) Confocal microscopic images of endogenous Rab30 in WT or Rab30-depleted HeLa cells. (C) Confocal microscopic images of EmGFP–LC3 in Rab30 knockdown cells using siRab30#2 during GAS infection. Bars, 10 μm. (D) The percentages of cells harboring GcAVs were quantified. Data represent the result of >100 cells in terms of the mean value ± SD from 3 independent experiments. * *P* < 0.05.
(TIF)

S4 Fig Influence of Rab30 WT, QL and TN expression on GcAV formation. (A) Confocal microscopic images of GcAVs in cells that expressed mCherry-Rab30 WT, QL, or TN. (B) The percentages of GcAV-positive cells were quantified. Data represent the result of >100 cells in terms of the mean value ± SD from 3 independent experiments. * *P* < 0.05. ** *P* < 0.01.
(TIF)
