# Supplementary figures and images for: Golgi-Resident GTPase Rab30 Promotes the Biogenesis of Pathogen-Containing Autophagosomes
Source: PLoS One. 2016 Jan 15;11(1):e0147061. doi: 10.1371/journal.pone.0147061 (PMC4714835; doi:10.1371/journal.pone.0147061)

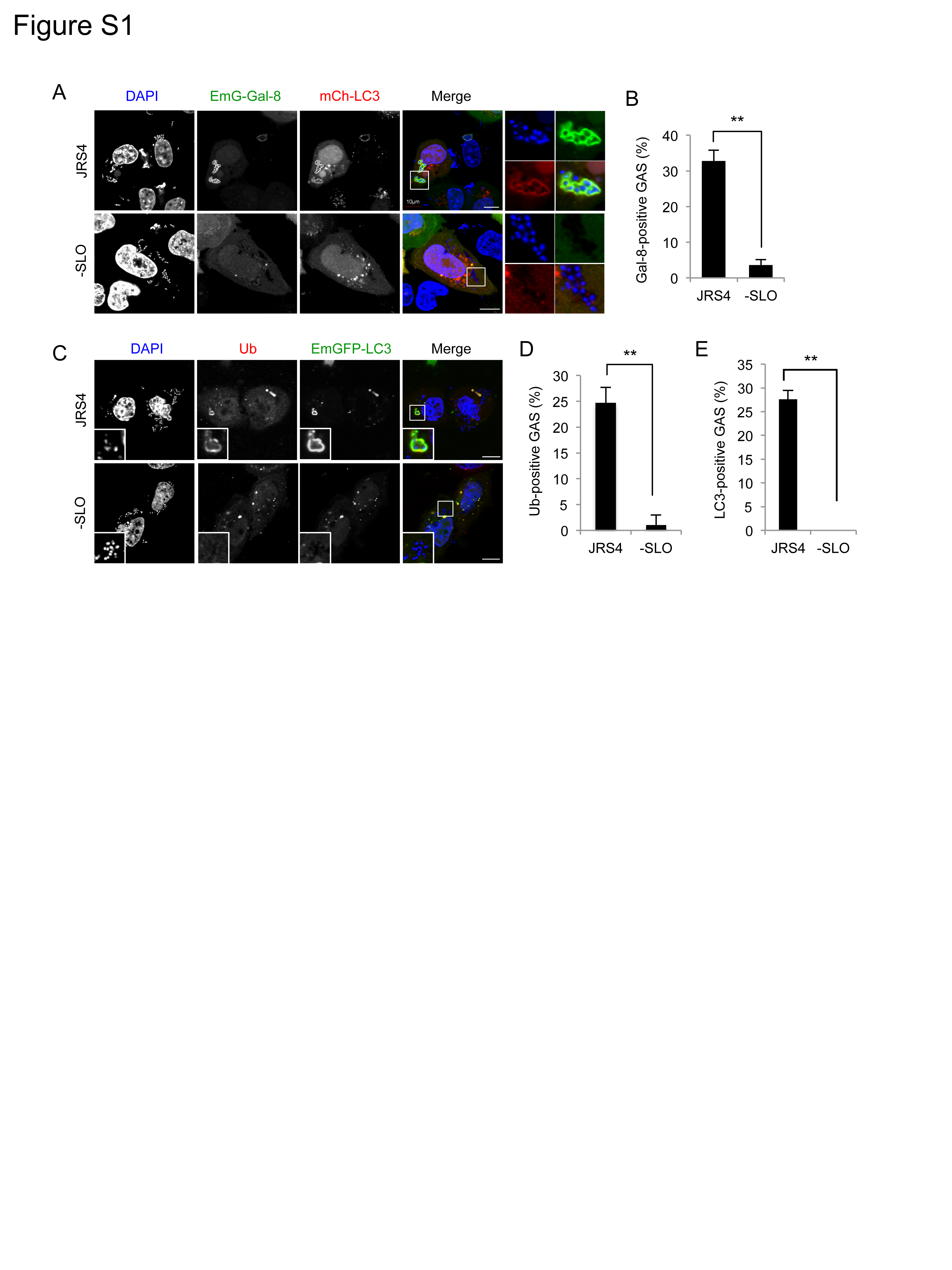

Supplement: S1 Fig — (A) Confocal microscopic images of EmGFP–Galectin-8 (Gal-8) with mCherry–LC3 in SLO-deficient GAS-infected cells. HeLa cells that expressed mCherry–LC3 and EmGFP–Gal-8 were infected with WT or SLO-deficient GAS for 4 h. Cells were then fixed, permeabilized, and stained for cellular and bacterial DNA with DAPI. Bars, 10 μm. (B) The percentages of cells with EmGFP-Gal-8-associated GAS were quantified. Data represent the result of >100 cells in terms of the mean value ± SD from 3 independent experiments. (C) Confocal microscopic images of EmGFP–LC3 and ubiquitin in SLO-deficient GAS-infected cells. HeLa cells that expressed EmGFP–LC3 were infected with WT or SLO-deficient GAS for 4 h. Cells were then fixed, permeabilized, and stained with anti-ubiquitin (Ub) antibody. (D, E) The percentages of cells with ubiquitin- (D) or LC3- (E) associated GAS were quantified. Data represent the result of >100 cells in terms of the mean value ± SD from 3 independent experiments. ** P < 0.01. (TIF) [file pone.0147061.s001.tif]

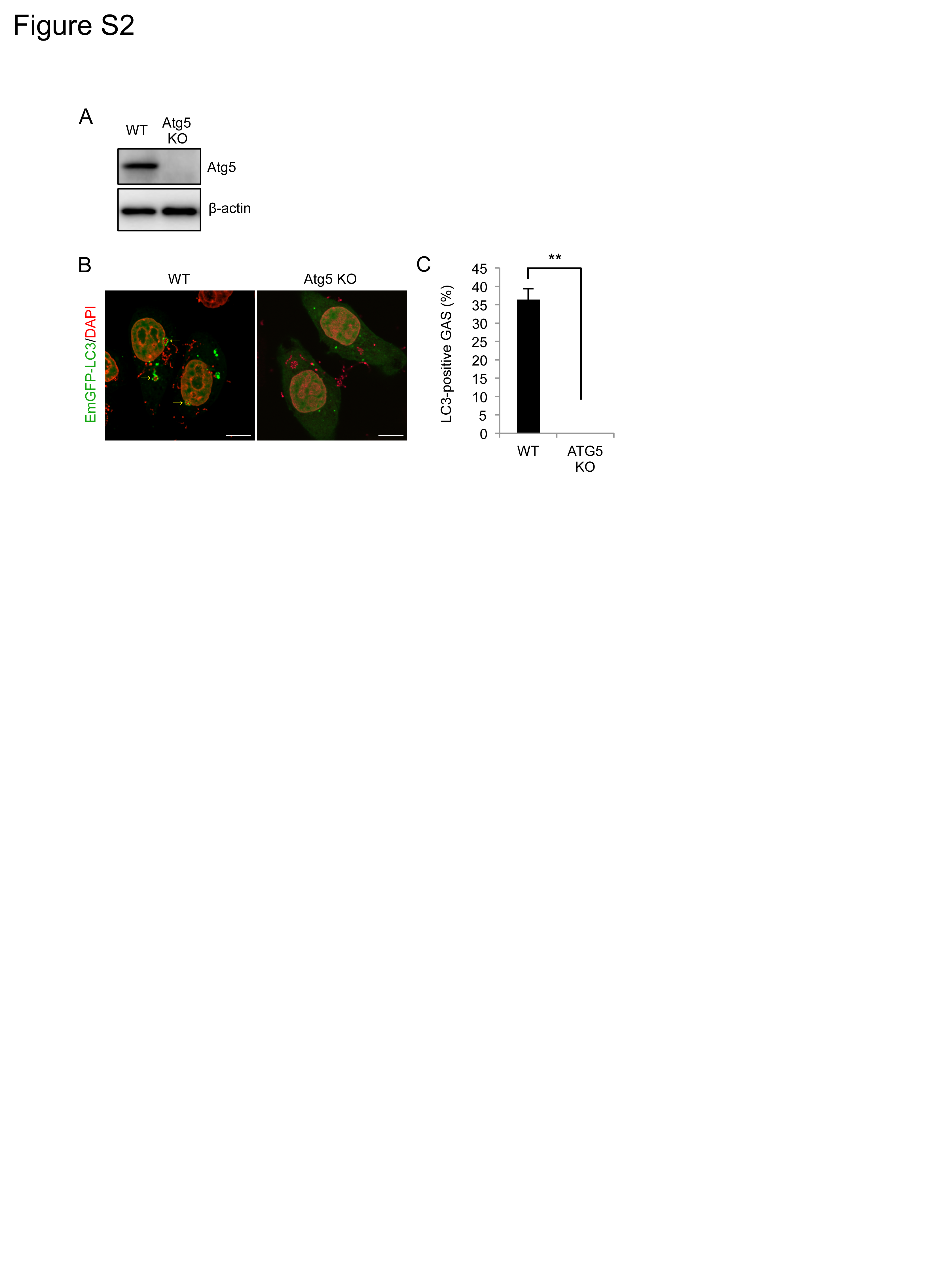

Supplement: S2 Fig — (A) HeLa WT or Atg5 knockout (KO) cells were analyzed by immunoblotting using anti-Atg5 antibody. (B) Confocal microscopic images of EmGFP–LC3 in Atg5 KO cells during GAS infection. HeLa WT or Atg5 KO cells that expressed EmGFP–LC3 were infected with GAS for 4 h. Cells were then fixed, permeabilized, and stained for cellular and bacterial DNA with DAPI. Bars, 10 μm. (D) The percentages of cells harboring GcAVs were quantified. Data represent the result of >200 cells in terms of the mean value ± SD from 3 independent experiments. ** P < 0.01. (TIF) [file pone.0147061.s002.tif]

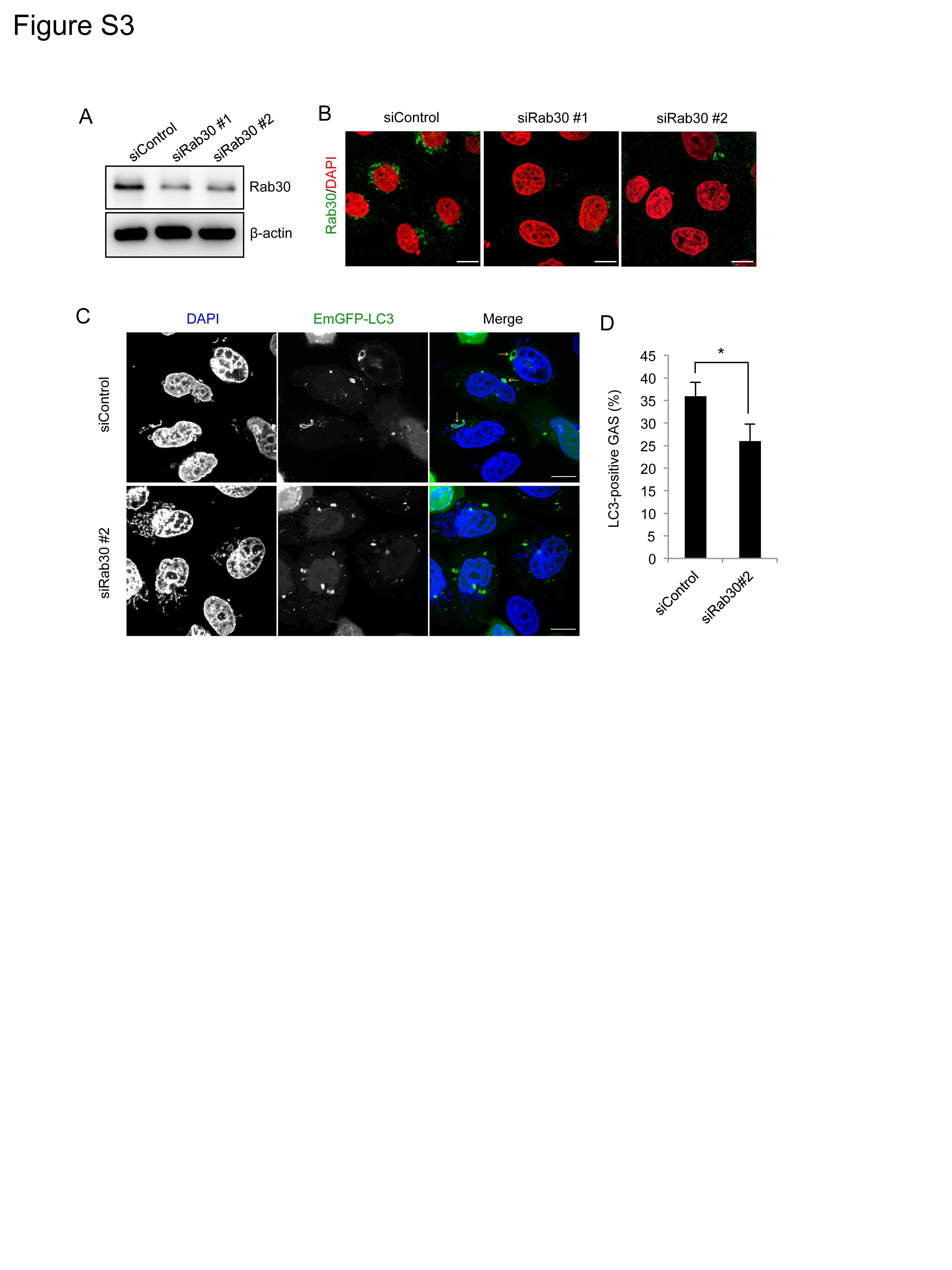

Supplement: S3 Fig — (A) HeLa transfected with siControl or siRab30 were analyzed by immunoblotting using anti-Rab30 antibody. (B) Confocal microscopic images of endogenous Rab30 in WT or Rab30-depleted HeLa cells. (C) Confocal microscopic images of EmGFP–LC3 in Rab30 knockdown cells using siRab30#2 during GAS infection. Bars, 10 μm. (D) The percentages of cells harboring GcAVs were quantified. Data represent the result of >100 cells in terms of the mean value ± SD from 3 independent experiments. * P < 0.05. (TIF) [file pone.0147061.s003.tif]

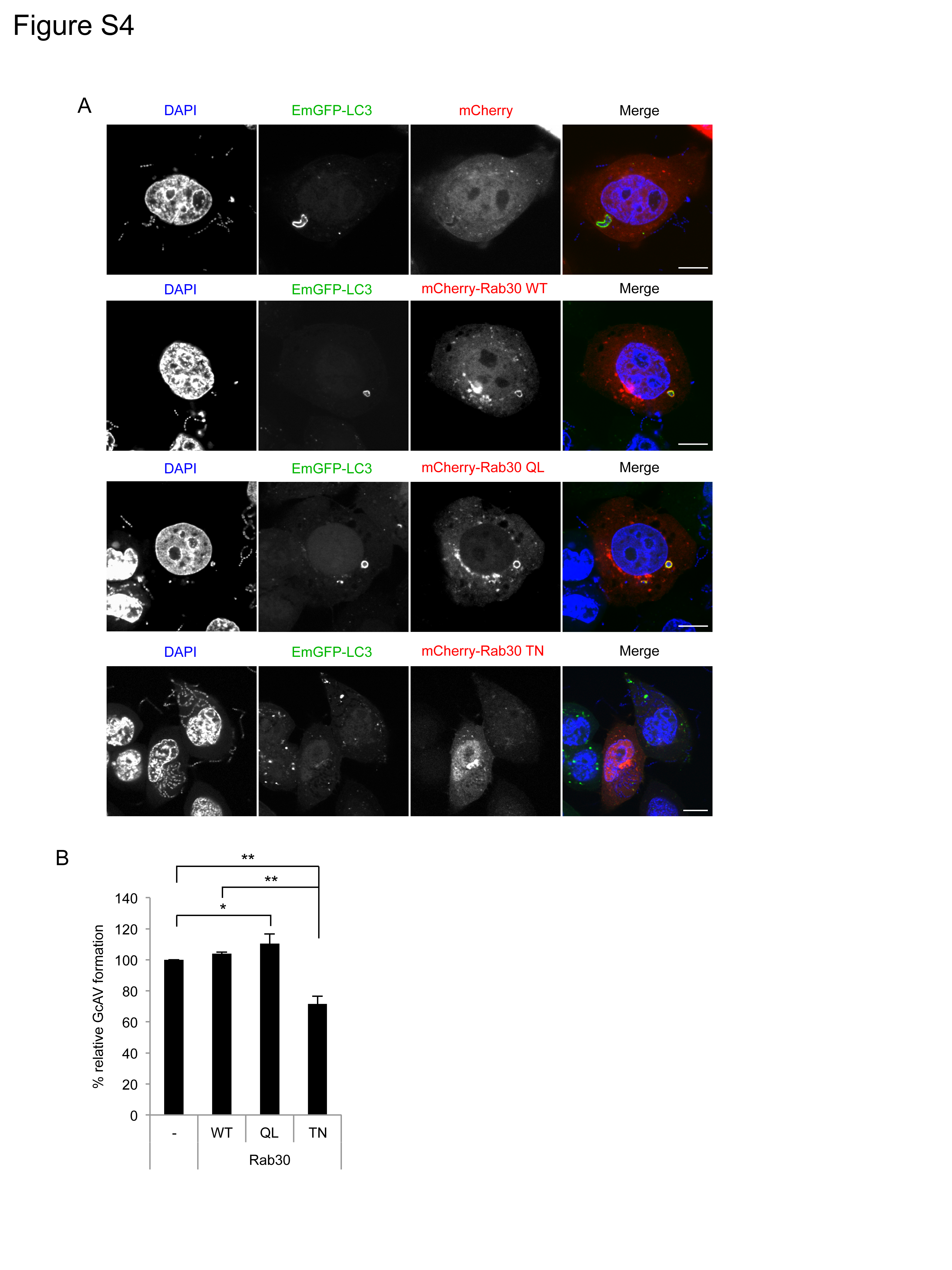

Supplement: S4 Fig — (A) Confocal microscopic images of GcAVs in cells that expressed mCherry-Rab30 WT, QL, or TN. (B) The percentages of GcAV-positive cells were quantified. Data represent the result of >100 cells in terms of the mean value ± SD from 3 independent experiments. * P < 0.05. ** P < 0.01. (TIF) [file pone.0147061.s004.tif]
